# Oral Manifestations of Sjögren’s Syndrome: Recognition, Management, and Interdisciplinary Care

**DOI:** 10.3390/medicina62010005

**Published:** 2025-12-19

**Authors:** Shu-Cheng Liu, Ming-Chi Lu, Malcolm Koo

**Affiliations:** 1 Department of Dentistry, Dalin Tzu Chi Hospital, Buddhist Tzu Chi Medical Foundation, Dalin 622401, Chiayi, Taiwan; 2 Division of Allergy, Immunology and Rheumatology, Dalin Tzu Chi Hospital, Buddhist Tzu Chi Medical Foundation, Dalin 622401, Chiayi, Taiwan; 3 School of Medicine, Tzu Chi University, Hualien City 970374, Hualien, Taiwan; 4 Department of Medical Research, Dalin Tzu Chi Hospital, Buddhist Tzu Chi Medical Foundation, Dalin 622401, Chiayi, Taiwan; 5 Dalla Lana School of Public Health, University of Toronto, Toronto, ON M5T 3M7, Canada

**Keywords:** dental disorders, Sjögren’s syndrome, xerostomia, interdisciplinary communication

## Abstract

*Background and Objectives*: Sjögren’s syndrome (SS) causes destructive salivary gland dysfunction with substantial oral morbidity. To synthesize practical, evidence-based approaches for early recognition, initial oral management, and timely referral to dental care. *Materials and Methods*: Narrative review of English-language literature from the Web of Science Core Collection and PubMed, prioritizing systematic reviews, randomized trials, and consensus guidelines. *Results*: Early oral signs include rapid multifocal root and cervical caries, burning sensations, and rising dental treatment needs. Unstimulated whole saliva ≤ 0.1 mL/min supports significant hypofunction and complements the 2016 ACR/EULAR criteria. Preventive care should combine dietary counseling, salivary stimulation, and topical remineralization. Adjuncts include high-fluoride toothpaste, biomimetic hydroxyapatite dentifrices, and casein phosphopeptide–amorphous calcium phosphate (CPP-ACP). However, evidence for fluoride varnish in SS remains mixed. Pharmacologic sialogogues require screening for contraindications. *Conclusions*: Embedding oral screening, simple salivary metrics, and a structured referral pathway into rheumatology visits can reduce preventable tooth loss and improve comfort, function, and treatment adherence.

## 1. Introduction

Sjögren’s syndrome (SS) is a systemic autoimmune disease characterized by chronic inflammation of exocrine glands, particularly the salivary and lacrimal glands. This process results in dryness of the mouth and eyes. Patients may also develop extra-glandular manifestations, including arthritis, vasculitis, and interstitial lung disease. SS predominantly affects women, with a female-to-male ratio of approximately 9:1, and the incidence peaks around 50 years of age. Autoantibodies such as anti-SS-related antigen A and B (anti-SSA/Ro and anti-SSB/La) are common serological markers, and focal lymphocytic sialadenitis is frequently observed in minor salivary gland biopsies. Given the potential involvement of multiple organ systems, multidisciplinary management is recommended for patients with SS [1,2].

Severe oral morbidity is a major and often debilitating component of Sjögren’s syndrome (SS), representing a critical, yet sometimes under-recognized, aspect of the disease’s total burden [3,4]. The resulting oral symptoms include chronic xerostomia (mouth dryness), burning sensations, altered taste, and dysphagia. Collectively, these manifestations profoundly diminish oral health-related quality of life [5,6]. For the rheumatologist, these are not merely localized dental issues. They significantly compromise nutrition, medication tolerance, and overall systemic health.

These oral manifestations are not just late-stage complications; they are often the earliest recognizable signs of SS. Compelling evidence shows that patients later diagnosed with SS have a significantly increased utilization of dental services for years prior to their formal rheumatologic diagnosis [7]. This finding reframes a patient’s dental history into a vital diagnostic opportunity, indicating the oral cavity as a key site for early disease detection.

**Figure 1:**
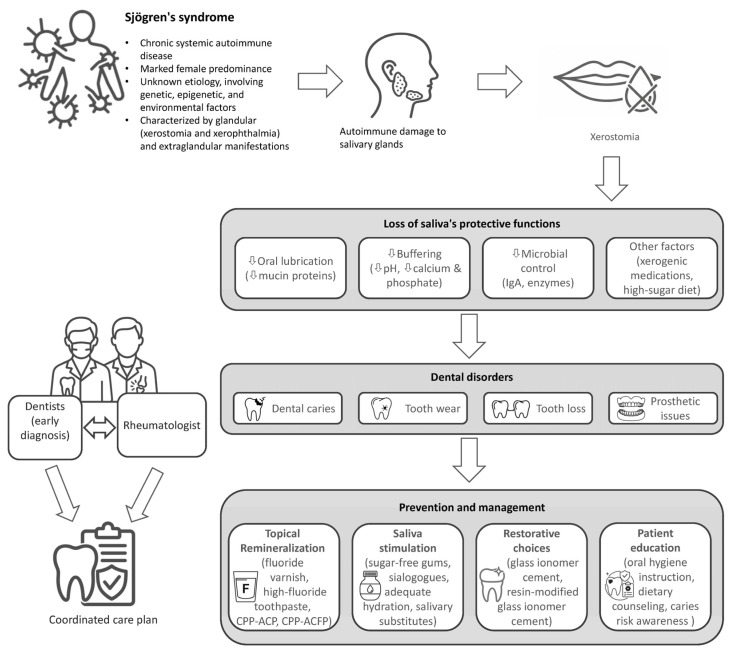
Pathophysiological cascade, oral manifestations, and multidisciplinary management of Sjögren’s syndrome. Sjögren’s syndrome is a chronic systemic autoimmune disease involving genetic, epigenetic, and environmental factors. It presents with glandular symptoms such as xerostomia and xerophthalmia, alongside various extraglandular manifestations. Autoimmune damage to salivary glands reduces flow and alters composition, compromising saliva’s protective functions, including lubrication, buffering, and antimicrobial action. Xerogenic medications and high sugar intake further increase oral disease risk. These changes predispose patients to caries, tooth wear, tooth loss, and prosthetic challenges. Effective management requires collaboration between dental and rheumatology teams. Key interventions include topical remineralization, saliva stimulation, suitable restorative materials, and patient education on oral hygiene, diet, and caries prevention. Horizontal arrows indicate disease progression, vertical arrows indicate downstream consequences culminating in prevention and management strategies, bidirectional arrows denote interdisciplinary collaboration, and converging arrows represent integration into a coordinated care plan.

Given the rheumatologist’s pivotal role in managing SS, this review focuses on three key actions for the routine visit: recognizing sentinel oral signs, initiating first-line measures, and referring efficiently to dental care. We address three gaps: (1) inconsistent recognition of early dental manifestations indicative of SS; (2) fragmented recommendations for topical remineralization and xerostomia management in cases of severe hyposalivation; and (3) limited implementation of interdisciplinary care pathways (Figure 1). To inform these recommendations, we conducted a narrative review of English-language literature published between January 2010 and July 2025.

## 2. Materials and Methods

A comprehensive search of Clarivate’s Web of Science Core Collection and PubMed for English-language literature published from January 2010 to July 2025 was conducted. The search strategy employed combinations of MeSH terms and keywords, including “Sjögren’s syndrome”, “oral manifestation*”, “xerostomia”, “salivary gland dysfunction”, and “dental disorder*”. The final search was conducted on 1 August 2025.

Inclusion criteria prioritized high-quality evidence, specifically systematic reviews, meta-analyses, randomized controlled trials, and clinical guidelines from rheumatology and dental associations. Where high-level evidence was limited, expert consensus statements and pivotal observational studies were included to provide clinical context. Articles were excluded if they were not available in English and were animal studies not directly applicable to clinical practice. The findings from this literature search are synthesized and presented thematically in the following sections: Epidemiology and Pathogenesis of Dental Disorders in Sjögren’s Syndrome (Section 3), Oral Manifestations in Sjögren’s Syndrome (Section 4), and Management of Oral Disease in Sjögren’s Syndrome: The Rheumatologist’s Role (Section 5).

During the preparation of this manuscript, the authors used ChatGPT (OpenAI; GPT-4o model) for the purposes of improving the readability and language of the manuscript. The authors have re-viewed and edited the output and take full responsibility for the content of this publication.

## 3. Epidemiology and Pathogenesis of Dental Disorders in Sjögren’s Syndrome

### 3.1. Epidemiology of Dental Disorders in Sjögren’s Syndrome

The prevalence and incidence rates of SS vary globally, primarily due to differences in diagnostic criteria and study designs. Recent genetic analysis further suggests that polygenic risk scores alone do not correlate with disease prevalence across European populations, indicating that environmental factors and diagnostic variability likely play a more significant role in these epidemiological discrepancies [8].

One systematic review and meta-analysis estimated the global pooled incidence rate for primary SS at 6.92 per 100,000 person-years, with an overall prevalence rate of 60.82 cases per 100,000 inhabitants. A notable female predominance was observed, reflected in a female-to-male ratio of 9.15 in incidence data and 10.72 in prevalence data. The overall mean age of individuals with primary SS was 56.16 years [9]. A population-based cohort study in the United States, utilizing physician diagnosis, found an age- and sex-adjusted prevalence of primary SS of 10.3 per 10,000. However, using stricter diagnostic criteria (the 2002 American–European Consensus Group [AECG] or 2012 American College of Rheumatology [ACR] criteria), the estimated prevalence decreased to 2.2 per 10,000 [10].

This systemic prevalence of SS carries significant dental ramifications. Studies consistently demonstrate that patients with SS experience considerably greater dental morbidity than individuals without the condition [4]. Multiple studies across diverse populations have quantified the oral health burden in patients with SS. A nationwide claim data-based cohort study conducted in Taiwan revealed a significantly increased prevalence (74.6% vs. 63.0%, *p* = 0.001) and frequency (median 5.37 vs. 1.45 per year, *p* < 0.001) of dental consultations among patients with primary SS relative to those without the disease. Notably, this increased utilization was evident for at least eight years prior to the formal rheumatologic diagnosis. Furthermore, the specific disease burden is severe: the risk of developing dental caries (adjusted incidence rate ratio [aIRR] 1.64, *p* < 0.001), pulpitis (aIRR 1.42, *p* < 0.001), gingivitis (aIRR 1.43, *p* < 0.001), periodontitis (aIRR 1.44, *p* < 0.001), oral ulceration (aIRR 1.98, *p* < 0.001), and stomatitis (aIRR 2.06, *p* < 0.001) was significantly higher among patients with primary SS [7].

Another case–control study found that patients with SS had a 61% higher risk of having experienced one or more dental extractions than age- and sex-matched patients without SS [11]. A cross-sectional study reported that patients with SS had more visible heavy dental plaque on teeth, a high prevalence of non-cavitated carious lesions, a large number of restored teeth, a high prevalence of cervical lesions, secondary caries, restoration failure, and a high percentage of wear lesions [12]. In addition to observational research, a genetic instrumental variable analysis also supports the detrimental effect of SS on dental caries and periodontitis [13]. In summary, while primary SS itself is relatively uncommon, affected individuals face a disproportionately high risk of dental diseases. This makes it critical for clinicians to recognize and address oral health within SS management.

### 3.2. Immunopathogenesis of Salivary Dysfunction

At the core of SS pathogenesis is the immune-mediated damage to exocrine glands. This process involves significant lymphocytic infiltration, primarily by T and B cells, which leads to chronic glandular inflammation, dysfunction, and eventual fibrosis [14]. For the rheumatologist, this familiar pattern of autoimmune attack has direct and severe functional consequences in the oral cavity. The resulting hyposalivation and altered composition compromise the mouth’s critical protective mechanisms. These include mechanical cleansing, acid buffering, antimicrobial action, and enamel remineralization [15].

This pathophysiological cascade creates a highly cariogenic environment. The loss of salivary pH buffering capacity, coupled with the loss of antimicrobial proteins, drives a profound dysbiotic shift. Research indicates a significant reduction in oral microbial diversity, specifically the depletion of healthy commensals such as *Neisseria* and *Hemophilus*. In their place, there is a marked proliferation of aciduric and cariogenic bacteria, specifically *Streptococcus mutans* and *Lactobacillus* species. This dysbiosis extends to fungi. A compromised mucosal barrier and lack of histatins favor *Candida albicans* colonization, making oral candidiasis a recurrent complication. These microbial changes can lead to the widespread and rapid destruction characteristic of the disease [16].

## 4. Oral Manifestations in Sjögren’s Syndrome

### 4.1. Oral Clues for the Diagnosis of Sjögren’s Syndrome

A patient’s oral health history often provides the earliest clues to SS. Rampant and aggressive dental caries are hallmarks, and their distribution patterns hold diagnostic value. Unlike typical dental caries, SS-related caries frequently occurs on atypical surfaces such as the roots, the gumline (cervical areas), and the incisal edges of anterior teeth [17]. This widespread and rapid destruction should raise concern for severe hyposalivation. A 72% higher odds of caries was reported in individuals with SS compared to those with dry mouth from other causes [18].

In addition to these clinical indicators, sialometry offers objective measures of salivary gland function. For routine clinical settings, a standardized 5 min collection of unstimulated whole saliva (UWS) is recommended. A flow rate of ≤0.1 mL/min indicates salivary hypofunction and aligns with one of the diagnostic criteria within the 2016 American College of Rheumatology/European League Against Rheumatism (ACR/EULAR) classification for primary SS [19].

However, clinicians should be aware of the limitations of this test. A prospective cohort study of 185 patients found that the threshold of ≤0.1 mL/min detected fewer than half of confirmed primary SS cases (sensitivity 43.0%, 95% confidence interval [CI] 32.8–53.7). This limited sensitivity persisted across subgroups, including women ≥ 50 years, where UWS performed especially poorly regardless of the cutoff used. Thus, the salivary test may be informative when low, but a normal result does not rule out the diagnosis [20].

To enhance test reliability and reduce false positives due to circadian variation, samples should be collected between 9:00 and 11:00 AM. Patients should be instructed to refrain from eating, drinking, smoking, or performing oral hygiene procedures for at least 90 min prior to the test. Moreover, reviewing and, when clinically appropriate, temporarily withholding xerogenic medications is critical for accurate interpretation [21].

Although rampant caries is a hallmark, soft tissue and periodontal changes often accompany hard tissue destruction. Patients with SS exhibit significantly higher rates of angular cheilitis, oral ulcerations, and atrophic mucosa compared to healthy controls [22]. Furthermore, the relationship between periodontal health and SS appears significant. A nationwide cohort study found that patients with chronic periodontitis had a substantially increased risk of developing primary SS diagnosis [23]. These findings suggest that persistent, unexplained periodontal inflammation may be an early indicator of the disease.

When patients report a “burning sensation” (stomatodynia), clinicians must differentiate between distinct etiologies, as management strategies differ significantly. Evidence suggests that burning mouth syndrome (BMS) and SS are distinct conditions that may present with analogous complaints [24]. Stomatodynia associated with SS is relatively uncommon and is frequently attributable to oral candidiasis. This should be suspected if the clinical examination reveals erythematous mucosa, removable white plaques, angular cheilitis, or atrophic depapillation. In contrast, BMS is a neuropathic condition characterized by persistent burning pain, commonly localized to the tongue, in the absence of visible mucosal lesions. Distinguishing these conditions is vital: candidiasis requires antifungal therapy, whereas BMS necessitates neuropathic pain management.

Clinicians should also recognize that these oral sequelae are driven primarily by the magnitude of hyposalivation rather than the classification of the disease. Secondary SS is distinguished by an associated connective tissue disease (e.g., rheumatoid arthritis, systemic lupus erythematosus). However, the resulting salivary hypofunction produces clinical manifestations, such as caries, mucosal atrophy, and susceptibility to candidiasis, that are indistinguishable from primary SS. Therefore, the preventive and management strategies outlined in this review apply equally to both subgroups.

### 4.2. Histopathologic Considerations: Differentiating Sjögren’s Syndrome from Granulomatous Diseases

The minor salivary gland biopsy (MSGB) remains a critical element in the diagnosis of SS, carrying significant weight in the ACR/EULAR classification criteria. The histopathologic hallmark of SS is focal lymphocytic sialadenitis, which involves dense aggregates of ≥50 lymphocytes localized around ducts and blood vessels. A positive biopsy for SS is defined by a focus score of ≥1 focus per 4 mm^2^ of glandular tissue [25].

However, clinicians must be vigilant for granulomatous diseases that can induce inflammation in the labial salivary glands, potentially mimicking or obscuring the diagnosis of SS. Unlike the lymphocytic foci of SS, these conditions are characterized by granuloma formation. Key differentials include the following: (1) sarcoidosis: This often presents with non-caseating epithelioid granulomas without the classic SS pattern of focal lymphocytic sialadenitis. Clinically, this may manifest as salivary gland swelling (e.g., Heerfordt syndrome) and must be distinguished from the glandular enlargement seen in SS [26]; (2) Crohn’s disease/orofacial granulomatosis: This can exhibit non-caseating granulomas that are often histologically indistinguishable from sarcoidosis on routine examination. Systemic evaluation is required to differentiate them, supported by clinical oral clues such as linear ulcers, cobble-stoning of the mucosa, or lip swelling [27]; (3) infectious granulomatous diseases: Conditions such as tuberculosis or atypical mycobacterial infections typically produce caseating granulomas with central necrosis, though non-caseating forms can occur. Special staining (e.g., Ziehl–Neelsen) is essential when infectious etiology is suspected [28]; (4) foreign-body granulomas: These may result from ruptured cysts or traumatic implantation of material. They are typically localized and contain foreign body giant cells, distinguishing them from the systemic autoimmune pattern of SS [29].

Accurate identification of these histopathologic patterns is vital to avoid misdiagnosis and to initiate appropriate therapy, as the management of granulomatous inflammation differs fundamentally from that of autoimmune sialadenitis.

### 4.3. Assessing the Impact of Oral Morbidity on Patient-Reported Outcomes and Systemic Health

The oral disease burden in SS directly and severely impacts patient-centered outcomes. Oral symptoms, including dry mouth, burning sensations, altered taste, and difficulties with speaking, eating, and swallowing, are major contributors to a diminished oral health-related quality of life [30]. The combination of rampant caries and pathological tooth wear, exacerbated by a lack of salivary lubrication and buffering capacity, often leads to irreversible tooth loss if not managed proactively [31]. Because dysphagia and oral pain can limit oral intake and medication tolerance, early control of oral symptoms is integral to maintaining nutritional status and adherence to systemic therapy.

This tooth loss is a significant adverse health outcome that impairs masticatory function and can compromise nutritional status [32]. Patients with SS experience substantially higher rates of missing teeth and complete edentulism compared to their non-SS counterparts. For example, a study using dental charts and radiographic data revealed a markedly higher prevalence of complete edentulism in the SS group (14.8%) versus the non-SS group (1.9%) [11]. A cohort study involving 205 female patients further demonstrated that patients with SS retained an average of only 21 teeth, compared to 24 in matched controls [33]. These findings are also supported by a meta-analysis of 21 studies including 3702 participants, which reported a mean tooth loss of 2.8 teeth (95% CI 2.05–3.50) in patients with SS relative to healthy individuals [34]. Furthermore, for patients who use dentures, the lack of saliva compromises suction and lubrication, often leading to poor fit, chronic pain, and ulceration [35]. These challenges are not just dental issues; they directly impact a patient’s eating ability, social confidence, and overall well-being.

### 4.4. Oral Health as a Factor in Systemic Disease Management

Poor oral health can significantly complicate the systemic management of SS. Chronic oral inflammatory conditions, such as periodontitis and oral ulcerations, which occur at a higher rate in patients with SS, can serve as a potential source of systemic inflammation or infection, a particular concern for patients receiving immunosuppressive therapies [36]. Furthermore, severe oral pain and dysphagia can negatively impact a patient’s ability to adhere to a regimen of oral medications [37].

The management of the dental disease itself creates a significant treatment burden. Standard dental fillings are highly susceptible to failure in the dry oral environment of SS, with one study showing a nearly threefold higher failure rate compared to controls [38]. This often initiates a frustrating and costly cycle of restorations and re-restorations that culminates in tooth extraction. Even advanced solutions like dental implants, while viable, require careful planning due to concerns about infection and potentially higher failure rates in this patient population [39]. This high burden of care shows the need for the rheumatologist to be an active partner in a proactive, interdisciplinary approach to prevent and manage the oral sequelae of the disease.

## 5. Management of Oral Disease in Sjögren’s Syndrome: The Rheumatologist’s Role

A successful management strategy for the oral manifestations of SS must be multifaceted and prevention-oriented, combining patient education, first-line symptomatic therapy, and timely dental referral (Table 1). These recommendations align with contemporary rheumatology guidance that emphasizes prevention, patient education, and coordinated interdisciplinary care [40,41].

### 5.1. First-Line Patient Counseling and Preventive Education

The rheumatologist’s role begins with empowering the patient through education and counseling on essential preventive strategies. This is a critical component of chronic disease self-management. Key counseling points include the following: (1) Dietary counseling: Patients should be advised to minimize frequent exposure to dietary sugars and acids, which significantly increase caries risk in a hyposalivatory environment [16]. (2) Lifestyle modifications: Patients should be advised to avoid alcohol and tobacco, as both can exacerbate oral dryness and increase the risk of dental disease [42]. (3) Meticulous oral hygiene: Emphasize the necessity of a strict oral hygiene regimen, including gentle brushing with a soft-bristled brush, fluoridated toothpaste after meals, and daily interdental cleaning to reduce plaque and prevent caries [41]. (4) Salivary stimulation and hydration: Patients should be encouraged to maintain proper hydration and to stimulate any residual salivary flow with sugar-free chewing gums or lozenges, particularly those containing xylitol [43].

### 5.2. Remineralization and Caries Control

Adults with SS are at very high caries risk. Daily 5000 ppm fluoride toothpaste or gel is recommended as the anchor measure, with instructions to apply a pea-sized amount twice daily, spit without rinsing, and reassess lesion activity at 3 months. This is feasible to prescribe from rheumatology clinics and is supported by contemporary professional guidance for high-risk adults [44].

Fluoride varnish can be offered as an adjunct in high-risk adults, yet SS-specific evidence is mixed regarding its added efficacy over daily high-fluoride toothpaste. A randomized trial involving 78 patients with SS reported no clear benefit compared with control. Therefore, varnish should be presented as an option rather than a blanket recommendation [45].

Biomimetic hydroxyapatite (HAP) dentifrices represent a suitable adjunct for patients with hyposalivation, particularly those seeking a low-fluoride option or those who may benefit from a dual-active regimen combining fluoride and HAP to target distinct pathways of enamel repair. Recent randomized trials and meta-analyses have demonstrated that HAP is non-inferior to fluoride toothpaste in preventing caries and promoting remineralization of early lesions, with favorable tolerability [46,47].

On the other hand, evidence is inconsistent for casein phosphopeptide–amorphous calcium phosphate (CPP-ACP) formulations. A meta-analysis suggests that it can improve remineralization of early enamel lesions compared with fluoride varnish [48], whereas others indicate that adding CPP-ACP to fluoride provides no additional benefit over fluoride alone [49].

### 5.3. Pharmacologic Management of Salivary Hypofunction

Clinical decision-making for managing hyposalivation should follow a stepwise approach. When patient counseling and local mechanical stimulation are insufficient to manage symptoms, the rheumatologist should consider escalating to systemic secretagogue therapy. This is most effective in patients with residual glandular function.

The primary pharmacologic agents for increasing salivary output are the systemic sialogogues pilocarpine and cevimeline [50,51]. Standard dosing for pilocarpine is typically 5 mg orally taken three times daily for at least 3 months. Cevimeline is generally dosed at 30 mg orally three times daily for at least 3 months [52]. If there is no subjective or objective improvement after treatment, the medication should be discontinued to avoid unnecessary polypharmacy and side effects.

When prescribing these medications, it is essential to counsel patients on potential side effects, such as nausea and excessive salivation, and to screen for contraindications, including uncontrolled asthma or chronic obstructive pulmonary disease (due to increased bronchial secretions), narrow-angle glaucoma (due to increased intraocular pressure), and acute iritis [52,53].

It is also important to manage patient expectations, as the evidence for many dry mouth therapies is limited. A Cochrane review of 36 randomized controlled trials involving 1597 participants found no strong evidence that topical treatments, such as oxygenated glycerol triester spray, gum chewing, and integrated mouthcare systems, are effective for relieving dry mouth symptoms [54]. Similarly, another Cochrane review of nine studies on non-pharmacological interventions for xerostomia concluded that there is low-quality evidence that acupuncture can improve dry mouth symptoms in patients who have undergone radiotherapy. The review also found insufficient evidence to determine the effects of electrostimulation devices on dry mouth symptoms or saliva production in patients with SS [55]. Where high-quality evidence is limited, shared decision-making that prioritizes symptom relief, safety, and feasibility is appropriate. Re-evaluate response within 4–6 weeks and discontinue ineffective therapies. A summary of these pharmacologic interventions, alongside the preventive and restorative strategies discussed throughout this review, is provided in Table 2.

### 5.4. Facilitating Effective Interdisciplinary Care: The Dental Referral

A structured referral to a dental professional is not a handoff but the initiation of a collaborative care partnership. The rheumatologist’s referral should include the SS diagnosis and duration, sialometry results, current systemic therapies, recent antibiotic or antifungal use, and any suspected candidiasis or mucosal pain. The main goals of this interdisciplinary care include establishing a rigorous preventive plan, managing existing dental disease, and evaluating for advanced prosthetic solutions. First, the cornerstone of dental management is intensive fluoride therapy to remineralize tooth structure and lower the incidence of new caries [56].

Second, the rheumatologist should be aware that standard dental restorations are highly susceptible to failure in patients with SS [38]. Understanding this helps the rheumatologist advocate for specific care pathways. While the technical selection of materials lies with the dental professional, the rheumatologist can support the patient by reinforcing the need for materials like glass ionomer cements, which release fluoride and chemically bond to tooth structure. Their effectiveness has been noted in case reports [57,58]. This is attributed to two key properties: sustained fluoride release for ongoing caries prevention and chemical adhesion to the tooth structure [59].

For extensive cervical or root lesions, resin-modified glass ionomer cement (RMGIC) may balance fluoride release with improved mechanical properties [60]. However, a definitive consensus on the most appropriate material for treating root caries remains elusive, with both a systematic review of 42 articles [61] and an umbrella review of 13 articles [62], indicating the need for further research. For cases of extended tooth tissue loss where direct fillings are insufficient, full-coverage crowns are often the definitive solution, as they provide a more durable and protective seal against the cariogenic environment [63].

Third, for patients with significant tooth loss, fixed solutions such as dental implants are generally preferred over removable dentures, which are often poorly tolerated due to mucosal dryness and discomfort [35,64]. However, implant therapy requires careful patient selection and perioperative planning due to potential risks of infection and failure in this population. Meticulous hygiene instruction and maintenance intervals should be ensured. Moreover, if removable dentures are the only option, modifications such as softer liners, frequent adjustments, and the use of water-soluble adhesives and moisturizers can enhance patient tolerance and function [65].

Effective management requires ongoing communication between the rheumatologist and the dental team. By actively participating in the management of oral disease, the rheumatologist can help mitigate one of the most significant sources of morbidity in SS and substantially improve the patient’s quality of life.

## 6. Critical Appraisal of Evidence and Limitations

### 6.1. Synthesis of Evidence

A critical synthesis of the reviewed literature reveals a divergence in the strength of evidence between disease epidemiology and clinical management. The epidemiological association between SS and adverse oral outcomes is robust, supported by large-scale, nationwide cohort studies demonstrating consistent hazard ratios for caries and periodontal disease. These findings are consistent across diverse populations, reinforcing the systemic nature of the oral burden. In contrast, the evidence base for specific dental management strategies in SS is less definitive and often relies on extrapolation from studies of radiation-induced xerostomia or general high-caries risk populations. Few randomized controlled trials are exclusive to the SS population, and those that exist often suffer from small sample sizes and heterogeneity in outcome measures. This has led to discrepancies in the literature, particularly regarding adjunctive therapies.

Consequently, current clinical practice relies heavily on expert opinion and lower-level evidence. There is a clear need for adequately powered, syndrome-specific comparative effectiveness trials to support the development of rigorous, evidence-based dental care guidelines.

### 6.2. Study Limitations

The findings of this review should be interpreted in light of several limitations. First, as a narrative review, this work did not employ the rigid systematic search protocols or formal quality assessment tools characteristic of systematic reviews. Consequently, despite our efforts to prioritize high-quality sources, the potential for selection bias cannot be fully excluded.

Second, the included literature shows substantial heterogeneity. Diagnostic criteria for SS evolved during the review period (from the 2002 American–European Consensus Group [AECG] criteria to the 2016 ACR/EULAR criteria), potentially creating variability in the patient populations described across studies.

## 7. Conclusions

Oral disease is a central and manageable component of SS morbidity. To address this burden, rheumatology teams should adopt a “screen–measure–refer” pathway: conducting a brief symptom check, measuring unstimulated salivary flow, and initiating timely referrals to dental colleagues.

Effective care requires a network of providers, including general dentists for routine prevention, and periodontists or oral medicine specialists for complex soft tissue and salivary management. This collaboration must be bidirectional; rheumatologists should advocate for their patients’ oral health, while dental providers must communicate oral findings that may signal systemic progression.

Prevention should prioritize daily high-fluoride toothpaste, with HAP as an adjunct where appropriate, targeted dietary counseling, and salivary stimulation. Looking ahead, priorities include SS-specific comparative trials of topical regimens, development of longer-retentive saliva substitutes, evaluation of the oral effects of biologics, and cost-effective referral models that fit routine rheumatology practice. Integrating these steps into multidisciplinary care can improve quality of life and reduce the long-term oral burden of SS.

## Figures and Tables

**Table 1:** A guide for the rheumatologist: Screening, initial management, and referral for oral manifestations of Sjögren’s syndrome.

Clinical Observations & Considerations	First-Line Action	Criteria for Dental Referral
Patient frequently sips water and may wake at night needing to drink Eating dry foods is difficult On exam, tongue depressor adheres to buccal mucosa; minimal saliva pooling; “lipstick sign” present (adherence of lipstick to the labial surface of anterior teeth due to lack of saliva film)	Counsel on hydration, sugar-free chewing gum/lozenges (xylitol-containing), and use of salivary substitutes Advise avoidance of caffeine, alcohol, and tobacco Consider systemic sialogogues (pilocarpine, cevimeline) after evaluating contraindications and side effects	Routine: All patients with confirmed Sjögren’s syndrome should establish a baseline assessment and preventive plan
Reports increased dental work in recent months Exam shows obvious dental caries, often at the gumline or incisal edges of anterior teeth; fractured teeth or failing restorations may be present	Reinforce strict oral hygiene and dietary counseling (reduce sugar/acid frequency) Emphasize the link between SS and high caries risk Recommend professional and prescription-strength fluoride	Urgent: Signs of rapid caries progression, dental pain, or suspected oral infection
Avoids certain foods due to oral pain Dentures have become loose or uncomfortable Exam reveals red, atrophic, or ulcerated mucosa; angular cheilitis; possible oral candidiasis (white plaques)	Assess for nutritional impact and weight loss Advise use of water-soluble lubricants for mucosa and dentures If candidiasis suspected, consider topical or systemic antifungals	Urgent: If oral pain interferes with adequate nutrition or medication intake Routine: For evaluation of denture fit and consideration of alternative prosthetic options such as implants

**Table 2:** Summary of preventive and management strategies for oral health in Sjögren’s syndrome.

Intervention	Recommended Frequency	Clinical Role & Evidence Level
Caries Prevention		
High-fluoride toothpaste (5000 ppm)	Twice daily	Anchor Measure. Supported by professional guidelines for high-risk adults; considered the standard of care.
Fluoride varnish (5% NaF)	Every 3 months (Professional)	Adjunct Option. Evidence in SS-specific trials is mixed; recommended based on individual caries activity rather than universal application.
Hydroxyapatite (HAP) dentifrice	Twice daily	Alternative/Adjunct. Recent trials suggest non-inferiority to fluoride; suitable for patients preferring fluoride-free options.
CPP-ACP formulations	Daily/As needed	Adjunct. Evidence is inconsistent regarding added benefit over fluoride alone; may aid remineralization of early lesions.
**Salivary Stimulation & Hydration**		
Mechanical stimulation (sugar-free gum/lozenges)	As needed	First-Line Symptomatic. Xylitol-containing products preferred to reduce caries risk; effective for transient relief.
Systemic sialogogues (Pilocarpine, Cevimeline)	Prescribed regimen (e.g., TID/QID)	Second-Line Therapeutic. Effective for increasing flow; requires screening for contraindications.
**Restorative Approach**		
Glass Ionomer/RMGIC	As required for lesions	Preferred Material. Releases fluoride and chemically bonds to tooth structure; lower failure rate than composite resin in dry mouths.

CPP-ACP: casein phosphopeptide–amorphous calcium phosphate; QID: four times daily; RMGIC: resin-modified glass ionomer cement; TID: three times daily.

## Data Availability

No new data were generated with this research.

## Abbreviations

The following abbreviations are used in this manuscript:

ACR	American College of Rheumatology
AECG	American–European Consensus Group
aIRR	Adjusted incidence rate ratio
anti-SSA/Ro	Anti-Sjögren’s syndrome-related antigen A autoantibodies
anti-SSB/La	Anti-Sjögren’s syndrome-related antigen B autoantibodies
BMS	Burning mouth syndrome
CI	Confidence interval
CPP-ACP	Casein phosphopeptide–amorphous calcium phosphate
EULAR	European League Against Rheumatism
HAP	Hydroxyapatite
MSGB	Minor salivary gland biopsy
QID	Four times daily
RMGICs	Resin-modified glass ionomer cement
SS	Sjögren’s syndrome
TID	Three times daily
UWS	Unstimulated whole saliva
